# Home-field advantage effect weakened over time but was strengthened by labile carbon input in later litter decomposition stage

**DOI:** 10.3389/fpls.2025.1545311

**Published:** 2025-03-14

**Authors:** Yafang Xue, Ning Ma, Lei Jiang, Weimin Wang, Shenggong Li

**Affiliations:** ^1^ National Ecosystem Science Data Center, Key Laboratory of Ecosystem Network Observation and Modeling, Institute of Geographic Sciences and Natural Resources Research, Chinese Academy of Sciences, Beijing, China; ^2^ College of Resources and Environment, University of Chinese Academy of Sciences, Beijing, China; ^3^ State Key Laboratory of Subtropical Silviculture, Zhejiang Agriculture and Forestry University, Hangzhou, China; ^4^ Shenzhen Ecological Environmental Monitoring Center of Guangdong Province, Shenzhen, China

**Keywords:** litter decomposition, fungi, community similarity, labile carbon, rhizosphere priming effect

## Abstract

Home-field advantage (HFA) hypothesis proposes that leaf litter decays more rapidly in its original place than elsewhere owing to specific litter-field affinity. However, the HFA effect may vary over time and receive influences from other external factors, and it remains unclear whether the labile carbon (C) in root exudates influences the HFA effect during later decomposition stage. We aim to 1) elucidate how the HFA effect varies over time, 2) demonstrate how the HFA effect changes when stimulated by labile C at the later decomposition stage, and 3) explore how fungi affect the HFA effect. We conducted a reciprocal litter transplant experiment using two tree species, (*Pinus elliottii* and *Cunninghamia lanceolata*) with a two-phase design (early vs. late decomposition, plus glucose addition). We harvested the samples of soil and litter after decomposition for 1, 2, 4 and 6 months. Glucose (labile C) was added to soil after decomposition of 4 months. The HFA effect decreased over time, and the fungal community dissimilarity between home and away soils, especially Eurotiomycetes, affected variations in HFA. Additionally, glucose additions led to a significant increase of 15.19% in the HFA effect (*p*<0.05) during later decomposition stage, which was primarily associated with Sordariomycetes. Our findings implies that the HFA in litter decomposition was mainly associated with specific fungal taxa. Importantly, the introduction of labile C strengthened the HFA effect at later decomposition stage. Therefore, it cannot be overlooked that the priming effect of labile C input on the HFA effect at later decomposition stage in future research. Our two-phase design study further highlights the differences in litter decomposition between home and away soils at different decomposing stages and the regulation of HFA by specific fungal taxa and labile carbon inputs, especially in the later decomposition stage.

## Introduction

Litter decomposition influences the carbon (C) balance between the biosphere and the atmosphere and regulates terrestrial C and nutrient cycles (Bradford et al., 2017). Litter decomposes faster in the soil beneath the plant that produces it (“home”) than underneath other plants (“away”), which is defined as the home-field advantage (HFA) (Gholz et al., 2000). Decomposers are “engines” that transfer C and nutrients from dead organic matter to living organisms (Ayres et al., 2009a; Wickings et al., 2012; Cao et al., 2024). They are more specialized in breaking down litter in the soil where they originate than other soils (Palozzi and Lindo, 2018; Maillard et al., 2023) because of adaptatively-formed specific litter-field affinity. Moreover, the affinity between litter and decomposer is time-dependent, which would provide further insights into the HFA effect (Li et al., 2020; Shigyo et al., 2024). Among these microorganisms, fungi play an important role in decomposing litter as they secrete various extracellular enzymes that allow them to break down complex recalcitrant components (Strickland et al., 2009). It is necessary to further investigate the fungal community composition involved in mediating the HFA effect of decomposing litter.

Litter composition varies during the decomposition process, with easily utilizable litter components (i.e., oligosaccharides and cellulose) decomposed first, followed by more recalcitrant constituents (i.e., lignin and polyphenols) (Berg, 2000, 2018; Kou et al., 2020). Correspondingly, the fungal communities in decomposing litter vary during decomposition (Voříšková and Baldrian, 2013). The affinity between litter and microbial community changes over time due to the variations in the decomposers and litter composition (Veen et al., 2019; Li et al., 2020; Luan et al., 2020), which further affected the HFA effect. Lin et al. (2019) demonstrated that litter from broadleaf, coniferous, and bamboo forests exhibited HFA effects in a reciprocal transplant experiment in subtropical forests. Previous study has shown that the HFA effect of grass decomposition tends to increase in the early stages of decomposition but then decreases in the later stages (Yuan et al., 2019). Nevertheless, the variation in HFA effects of coniferous leaf litter decomposition in subtropical forests remains underexplored. Additionally, it is reported that over a longer period, the HFA effect is more strongly regulated by the dissimilarity in fungal communities between home and away environments than by that of bacterial communities (Li et al., 2020). Although some studies have revealed the impact of fungal community influences on the HFA effect (Lin et al., 2019; Veen et al., 2019), they overlooked the influence of time and some external disturbances (such as the influence of root activity). Therefore, it remains necessary to monitor the temporal changes in fungal community composition and HFA effect using multiple destructive harvests, and to consider the impact of roots.

Litter decomposition is influenced by plant roots, which proliferate into the forest floor to obtain nutrients (Wang et al., 2016; Kou et al., 2018), and C from plant roots is transported to soil via root exudates and rhizodeposits (Berendsen et al., 2012). The HFA effects may be influenced by root activity, which could affect the release rates of C and nutrients from litter. This provides new insights into carbon and nutrient cycling associated with the HFA effects of litter decomposition. Plant-soil interactions in the rhizosphere mediate approximately 50% of the total CO_2_ released by terrestrial ecosystems (Hopkins et al., 2013). In particular, root exudates, consisting of 50-70% glucose, are easily utilized by heterotrophic microbes and thus accelerate the decomposition of old organic matter (Shahzad et al., 2015; Tian et al., 2019), a phenomenon referred to as the rhizosphere priming effect (Huo et al., 2017). Initially, labile C creates microbial community hotspots and stimulates the growth of *r*-strategists for a short time (0-3 days). When the labile C is exhausted, other microorganisms (mainly *K*-strategists) that can utilize less readily available substrates (e.g. soil organic matter) benefit from the residual necrotic clusters left after the depletion of readily available organics (Kuzyakov, 2010). The *K*-strategists becomes dominant, playing a key role in decomposing old organic matter (Fontaine et al., 2003; Kuzyakov, 2010). However, few studies have focused on the effect of labile C on litter decomposition and the HFA effect A field study demonstrated that local roots can maintain litter-field affinity and impact the HFA effect (Tian et al., 2018). This is likely because root exudates of native plants can positively influence the microbial community in decomposing native litter but not in foreign litter, enhancing the HFA effect (Badri and Vivanco, 2009; Tian et al., 2018). When natural polymers decompose, they typically release monosaccharides into the soil. Additionally, glucose is predominantly found among the sugars released in rhizodeposits (Kuzyakov, 2010; Bastida et al., 2019; Chen et al., 2019). Microbes primarily consume endogenous C in litter during the early decomposition stage (Berg and McClaugherty, 2014). As these resources deplete, the litter decomposition rate quickly slows down, shifting to the later decomposition stage. During this stage, exogenous C input provides additional energy to microbes. Labile C (glucose) may further strengthen the relationship between litter and microorganisms from the same environment, compared to those from the different environments. Zheng et al. (2022) reported that the priming effect of glucose modified the HFA effect of absorptive and transport root litter decomposition; however, the impact of glucose on the HFA in leaf litter during later decomposition stages remains unclear.

Plantation forests contribute significantly to the forest C sink (Piao et al., 2009), especially in subtropical Asia (Yu et al., 2014). This study examined the variation in the HFA effect of litter decomposition over time using the litter from *Pinus elliottii* and *Cunninghamia lanceolata* plantations in subtropical China. We aimed to determine whether glucose additions affect the HFA glucose additions affect the HFA during the later decomposition stages and how the soil fungal community composition mediates the HFA, which is still unclear. Furthermore, we performed a reciprocal transplant microcosm experiment containing these two tree species with a two-phase design (early vs. late decomposition, plus glucose addition) and tested the following three hypotheses: (1) fungal community composition varies over the stages of litter decomposition; (2) fungal community composition affects the HFA effect; and (3) at later decomposition stage, glucose addition alters the fungal community composition and enhances the HFA effect, which is mediated by the fungal taxa. To test hypotheses 1 and 2, we collected the remaining litter and soil after decomposition for 1, 2, 4, and 6 months. To test hypothesis 3, we added glucose after litter decomposition for 4 months, harvested the litter after decomposition for another 2 months, and compared the HFA with and without glucose additions.

## Materials and methods

### Litter and soil collection

This study was conducted at the Qianyanzhou Ecological Station, Chinese Academy of Sciences, Jiangxi Province, southeastern China (26°44′ N, 115°03′ E). According to the USDA soil classification system, the soil type is Inceptisol developed from weathered red sandstone and mudstone (Wang et al., 2012). The continental subtropical monsoon climate in the region is characterized by the simultaneous occurrence of rain and heat, with an average annual precipitation and temperature of 1,475 mm and 17.9°C, respectively (Wen et al., 2010).

In this study, newly senesced leaf litter of *P. elliottii* and *C. lanceolata* was collected with litterfall traps between October and December 2020 from their respective plantation stands for the litter decomposition experiments as these two tree species are dominant for vegetation restoration in the region. The collected leaf litter was evenly mixed separately for each litter species, cut into 1-cm fragments, and oven-dried at 60°C until constant mass. In December 2020, we selected four mature, healthy individual trees of each species with similar diameters for collecting soil samples. Soil samples were collected at the soil depth of 0-20 cm from each of these two plantations, and frozen using ice in the field and transported to the laboratory. The soil samples were pulverized and sieved through a 2 mm soil sieve. They were taken as the standard soils to avoid confounding effects (Gundale et al., 2017) and then used to determine whether the fungal community composition affects litter decomposition differently.

### Microcosm decomposition experiment

We used a reciprocal transplantation design for litter decomposition. Both *P. elliottii* and *C. lanceolata* litter were decomposed in soils of the two tree species, i.e in home and away soils. The soils were placed in polyethylene terephthalate containers with a height of 6.5 cm and diameter of 8.5 cm. A sample of 100 g soil was placed in each container (microcosm). Next, we placed a litter bag (1-mm mesh) with 1.6 g of litter on the soil in each microcosm and placed the microcosms in a dark incubator at 25°C. The microcosm experiment comprised 80 containers (2 litter species × 2 soil sources × 5 harvests × 4 replicates). Samples were collected four time (1, 2, 4, and 6 months). The 6-month samples, collected in two batches (with and without glucose addition), were considered the final harvest and counted as two sampling events, totaling five samplings. Water was added to the soil every fortnight to maintain the soil moisture at 60% of its water-holding capacity. Small holes were made in the lids of the microcosms for aeration and to minimize water loss from the interior.

We divided the decomposition experiment into two phases (early and late stages) to test our hypotheses. The phase I experiment addressed how fungal community composition and HFA effects change over time. Leaf litter and soil samples were harvested after 1, 2, and 4 months of decomposition; in total, 48 containers were sampled. The remaining 32 containers were equally divided into two groups (16 containers per group) for the phase II experiment to determine how the glucose additions affected the fungal community composition and HFA effect during late decomposition stages. Glucose was added to one group, and water was added to the other as the control (CK). Referring to the previous study on the decomposition of mixtures of leaves and roots (the mass of leaf: root = 2:1) (Jiang et al., 2019), we calculated the glucose addition rate according to the mass of root was 0.8 g (the mass of leaf litter was 1.6 g in our study). The glucose addition rate was determined based on the finding that root exudates constitute 30% of the total organic carbon content in roots, as reported in a global dataset on belowground carbon allocation, and more details see a previous study (Zheng et al., 2022). We added the glucose solution (8 mL, 3.4%) to the soil surface, which was in direct contact with litter bags. These soil and leaf litter samples were harvested after an additional 2 months of decomposition (i.e. 6 months since the initial decomposition). We added glucose to the soil surface in contact with litter bags after four months of decomposition for the following reasons: the mass loss of *P. elliottii* and *C. lanceolata* in the field during the early decomposition stage ranges from 25% to 40% (Jiang et al., 2018; Mao et al., 2021; Wang et al., 2022; Wu et al., 2022), after this period, the rate of decomposition slows down sharply, signifying the transition to the later decomposition stage. In our study, the mass loss of litter from *P. elliottii* and *C. lanceolata* was observed to be 31.59% and 42.26% after four months of decomposition. Thus, we consider that the litter decomposition entered the later stage of decomposition after being incubated in the microcosm for four months in this study.

When harvesting, we removed litter bags from the microcosms, carefully opened them to reduce litter loss, and dried the litter at 60°C. We then collected soil samples and froze them at –80°C for fungal community analysis.

The leaf litter decomposition rate was calculated as mass loss between sampling intervals or mass loss compared with the initial litter mass as shown in Equation 1:


(1) $$Mass\;loss\;\left(\%\right)=\frac{\left(M_{t_{0}}-M_{t_{\text{i}}}\right)}{M_{t_{0}}}\times 100\%$$

where *M*_t0_ and *M_t_*_i_ are the litter mass in the litter bag at the initial time *t_0_* and sampling time *t_i_*, respectively.

### Soil fungal community determination

We extracted 0.5 g from each soil sample using a PowerSoil kit (MO BIO Laboratories, Carlsbad, CA, USA). The primer sets ITS1F (5′-CTTGGTCATTTAGAGGAAGTAA-3′) and ITS2R (5′-GCTGCGTTCTTCATCGATGC-3′) were used to amplify the polymerase chain reaction (PCR). PCR products were separated on 2% agarose gels and purified using an AxyPrep DNA Gel Extraction Kit (Axygen Biosciences, Union City, CA, USA). Sequencing was conducted using the Illumina MiSeq PE300 platform (Illumina, San Diego, CA, USA) at Majorbio BioPharm Technology Co., Ltd. (Shanghai, China). To improve the read quality, we truncated any site in a 350-bp read with an average quality score of< 20 bp in a 50-bp sliding window. We discarded reads truncated shorter than 50 bp, overlapping less than 10 bp, and containing ambiguous characters. Chimeric sequences were removed using the UCHIME algorithm (Edgar et al., 2011). Operational taxonomic units (OTUs) were clustered at the level of 97% sequence similarity using the UPARSE program (Edgar, 2013). A random subset of 57,742 sequences per sample was used for analysis. We use FUNGuild to determine fungal functional groups, considering only the classification with ‘highly probable’ and ‘probable’ confidence levels, and exclude those that are unassigned. We classify fungal species as saprotrophic, pathotrophic and symbiotrophic fungi, where fungi that contain saprotrophic function were classified as saprotrophs.

### Data analysis

The HFA for mass loss was calculated as follows, as shown in Equation 2 (Ayres et al., 2009b):


(2) $${\text{A}}_{\text{RMLa}}\;\left(\%\right)=\frac{A_{a}}{A_{a}+B_{a}}\times 100$$

where *A*_RMLa_ represents the relative mass loss of litter A at site *a* and *A*_a_ and *B*_a_ represent the percentage mass loss of litter *A* and *B* decomposed at site *a*, respectively, as presented in Equation 3.


(3) $$\text{HFA }\left(\%\right)=\left[\frac{A_{RMLa}+B_{RMLb}}{2}/\frac{A_{RMLb}+B_{RMLa}}{2}\right]\times 100-100$$

where HFA indicates the percentage faster mass loss of litter when decomposing at the native versus the foreign site and is a net value for the litter of both species in the reciprocal transplant experiment.

To improve the HFA assessment for each species, according to Equation 4, the mean HFA (percent increase in mass loss at the home site compared with that of the away site) was used (Austin et al., 2014):


(4) $$\text{Mean HFA }\left(\%\right)=\left(ML_{home}-ML_{away}\right)/ML_{away}\times 100$$

where *ML_home_* and *ML_away_* are the mass loss of a given species at the home and away sites, respectively.

To determine the variation in fungal community composition and HFA effect over time (hypotheses 1 and 2), we used data on litter mass and fungal community of soil samples without glucose addition. Meanwhile, we tested hypothesis 3 using data obtained from litter decomposition after 6 months of decomposition, which were with or without glucose added after 4 months.

To test the effect of soil source, litter species and time (referring to hypothesis 1 regarding the change in HFA over time) or treatment (referring to hypothesis 3 regarding the effect of glucose additions on HFA) on fungal alpha diversity, we employed an analysis of variance (ANOVA). Based on the Bray–Curtis distance matrix, principal coordinate analysis (PCoA) was used to visualize fungal community composition. Permutational multivariate analysis of variance (PERMANOVA) was used to determine how soil source, leaf species, and harvest time or treatment affected fungal community composition. We used R (R Core Team, 2020) to perform PCoA and PERMANOVA with the “*vegan*” package. The difference of HFA in decomposition after 1, 2, 4, and 6 months without glucose addition was evaluated using one-way ANOVA, which was also performed to determine the difference in mean HFA and the relative abundance of dominant fungal taxa at phyla or class level, corresponding to these intervals. Furthermore, a *t*-test was conducted to determine the differences in HFA, mean HFA, and the relative abundance of dominant fungal taxa at phyla or class level among samples collected at the end of the 6th month of decomposition, following the glucose addition or no addition at the end of the 4th month.

To improve the match between HFA and fungal dissimilarity, we used the mean HFA of the litter of each species. Regarding fungal dissimilarity, the whole community and dominant fungal taxa were determined by calculating the difference between home and away sites for each species. The Bray–Curtis dissimilarity index was used to determine the fungal community dissimilarity. According to Equation 5, the dissimilarity of fungal taxa or saprotrophs in abundance was calculated as follows (Veen et al., 2019):


(5) $$\text{dissimilarity abundance}=\left|log2\left(\frac{X_{iI}}{X_{jJ}}\right)\right|$$

where *X_iI_* and *X_jJ_* are the relative abundance of fungal taxa *X* or saprotrophs in soil *I* with litter *i* and in soil *J* with litter *j*, respectively.

To determine how fungi affect HFA over time, we first conducted a linear regression analysis with mean HFA as the dependent variable and fungal community dissimilarity or dissimilarity abundance in saprotrophs for the litter of each tree species as the predictor variable. Subsequently, a further linear regression analysis was executed, with mass loss as the dependent variable and the relative abundance of saprotrophic fungi as the predictor variable. Multiple regression analyses were performed with the mean HFA as the dependent variable and the dissimilarity abundance of the eight dominant fungal taxa as predictor variables. When the dissimilarity abundance of one fungal group could explain the mean HFA, the relationship between the mean HFA and fungal taxa dissimilarity was assessed with linear regression. The same approach was employed to determine how fungi affect HFA after adding glucose in the later decomposition stages.

These analyses were conducted using R version 4.0.5 (R Core Team, 2020). The normality and variance homogeneity of data were tested using the Shapiro–Wilk test and Levene’s test, respectively. Additionally, data were transformed where necessary to meet the assumptions.

## Results

### Variation in fungal community over time

**Figure 1:**
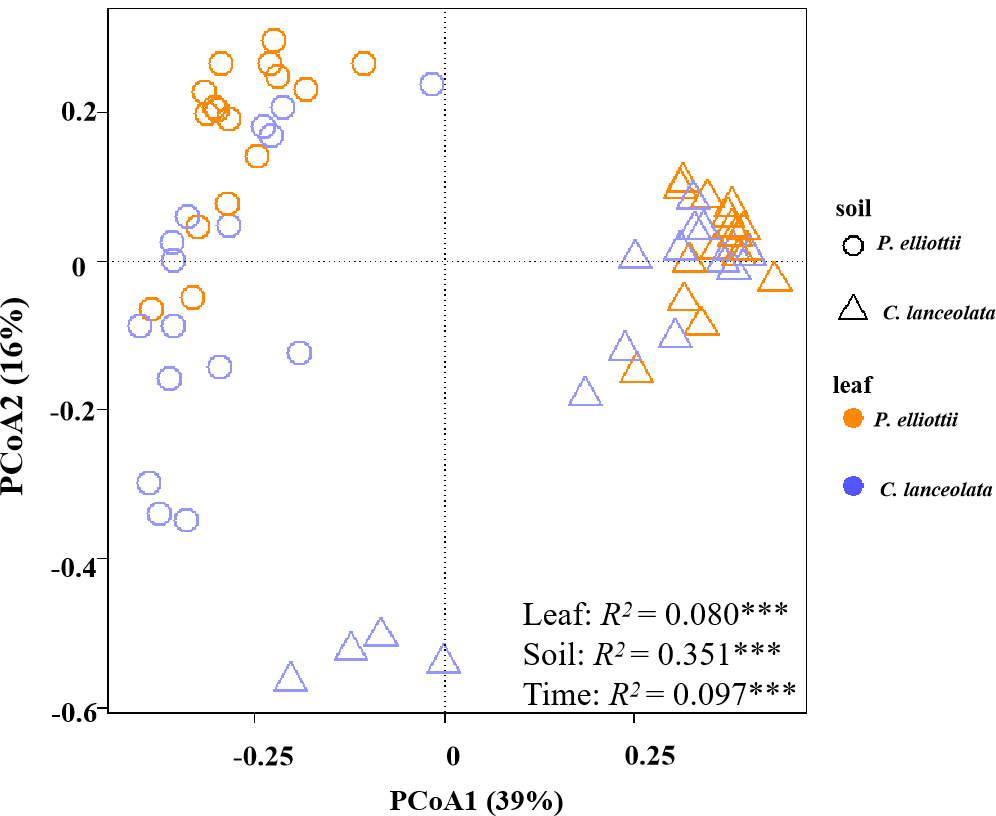
Principal coordinate analysis (PCoA) plot of fungal community composition in soils without glucose addition across four harvests. The compositional variation is represented by the Bray–Curtis distance matrix based on the abundance of operational taxonomic units (OTUs). Soil sources are indicated by different symbols, while leaf litter species are indicated by different colors. The asterisks ** and *** indicate P < 0.01 and P < 0.001, respectively.

**Figure 2:**
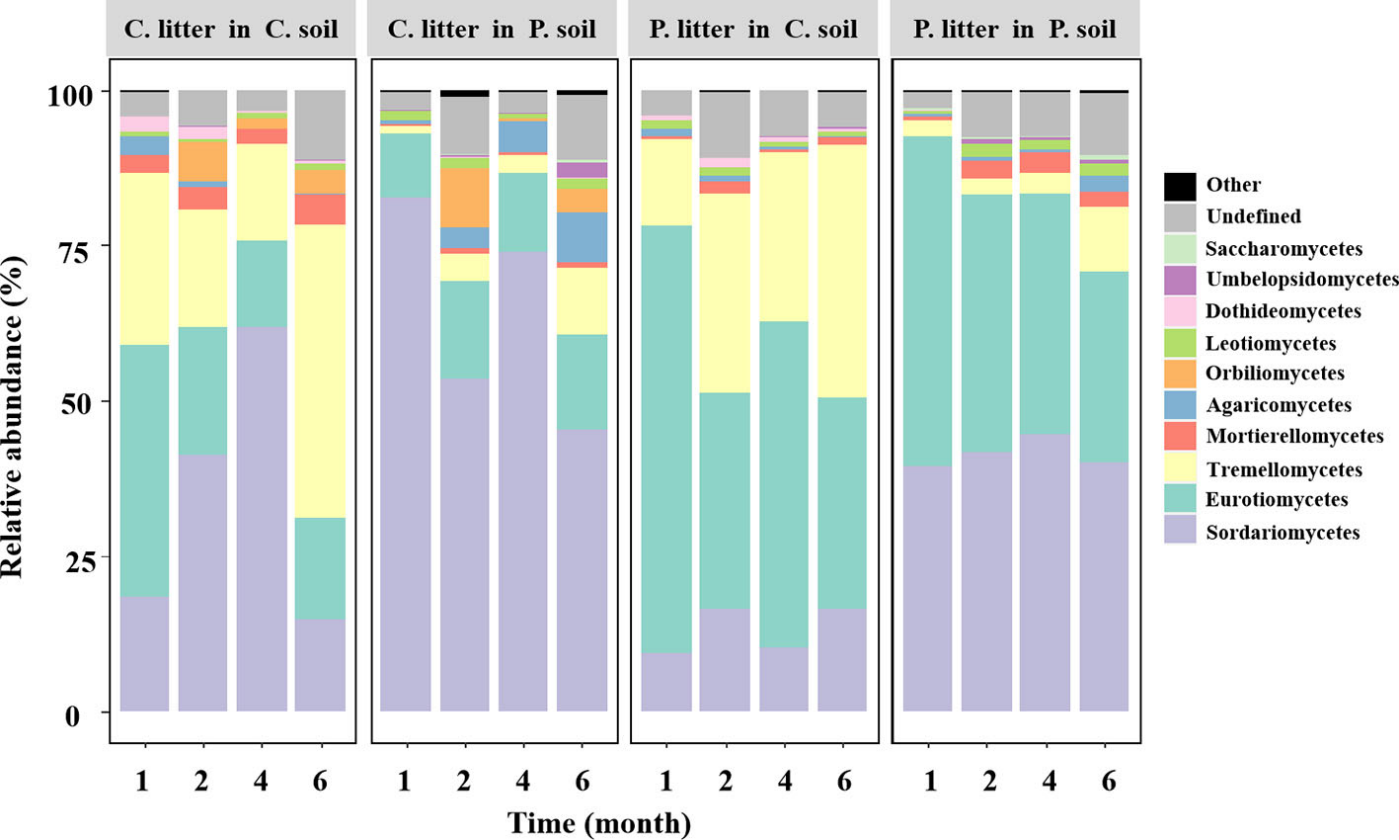
Variations in the relative abundance of the most abundant fungal classes in all soils without glucose addition over time. Different panels represent different soil sources and tree species, indicated by the names on the panel: P, *Pinus elliottii*; C, *Cunninghamia lanceolata*. Within each panel, individual bars represent different harvest times of decomposing litter.

Across all samples without glucose addition after 1, 2, 4, and 6 months of decomposition, the observed fungal species was significantly affected by soil source (*p*< 0.001) and time (*p* = 0.013), where the fungal Shannon diversity was significantly influenced by litter species (*p* = 0.022) and time (*p* = 0.008, **Supplementary Table S2**). The fungal community composition was significantly affected by the soil source, litter species, and harvest time (**Figure 1**; **Supplementary Table S3**). Soil source was the most important explanatory variable for fungal composition variation (35.1%), followed by harvest time (9.7%) (**Supplementary Table S3**). The relative abundance of the phylum Ascomycota tended to decrease over time, while that of Basidiomycota tended to increase (**Supplementary Table S4**). The dominant classes across all soil samples in the first experiment were Sordariomycetes, Eurotiomycetes, Tremellomycetes, Mortierellomycotina, Agaricomycetes, Orbiliomycetes, Leotiomycetes, and unclassified fungi, accounting for > 95% of the total fungal community (**Figure 2**; **Supplementary Table S5**).

### Variation in the HFA over time

**Figure 3:**
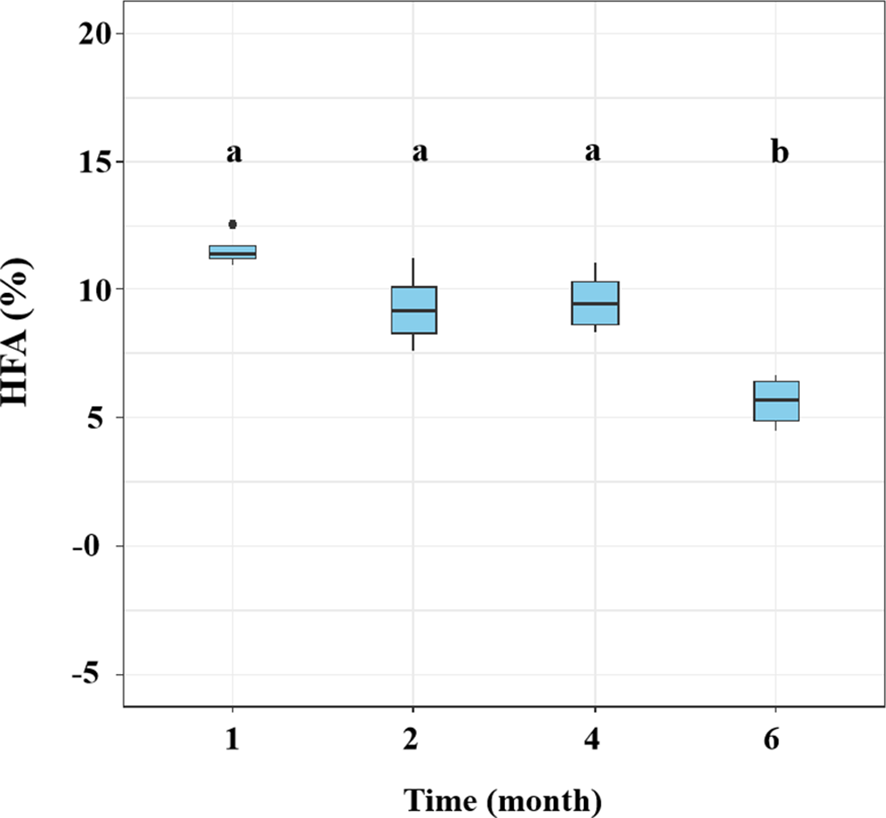
Variations in the home-field advantage (HFA) effect over time for leaf litter decomposition of *Pinus elliottii* and *Cunninghamia lanceolata* without glucose addition. Data are shown as mean ± SE. Bars topped with different letters indicate significant differences among harvest periods (Bonferroni *post-hoc* test, *p<* 0.05).

**Figure 4:**
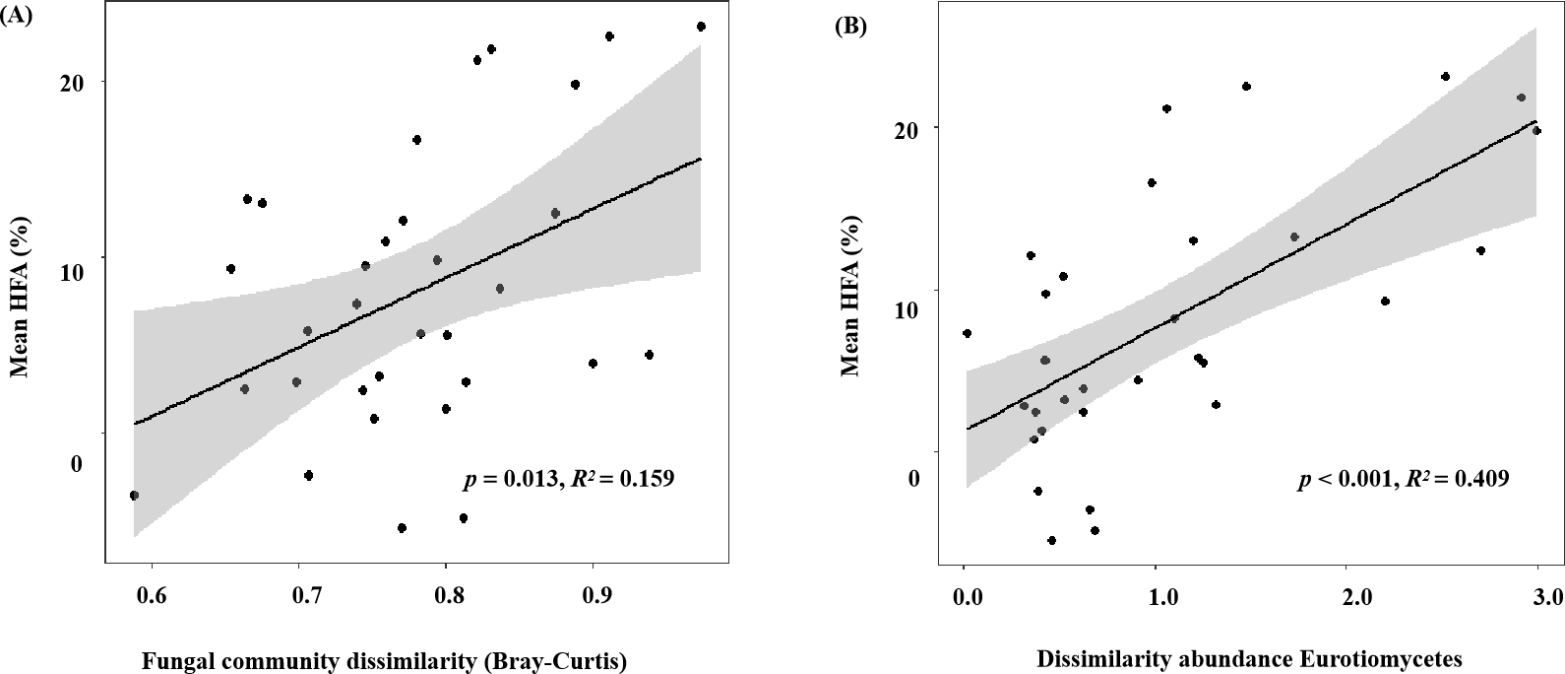
**(A)** Fungal community dissimilarity (Bray–Curtis) and **(B)** dissimilarity in the abundance of Eurotiomycetes (calculated as the logarithm of the ratio of the relative abundance of Eurotiomycetes in home and away soils) plotted against the mean home-field advantage (Mean HFA) effect. Sample data from the soils without glucose addition were collected at four harvest times (after decomposition of 1, 2, 4, and 6 months).

The HFA effect of litter mass loss was positive after 1, 2, 4, and 6 months of decomposition (**Figure 3**). No significant differences in HFA were observed after 1, 2, or 4 months of decomposition but HFA decreased significantly after 6 months of decomposition compared to that in the preceding 4 months (*p*< 0.05; **Figure 3**). During litter decomposition, the fungal community dissimilarity explained the magnitude of the mean HFA (*p* = 0.013, *R* ^2^ = 0.159; **Figure 4A**). Additionally, linear regression analyses revealed that the dissimilarity in the abundance of the class Eurotiomycetes (phylum Ascomycota) explained the magnitude of the HFA effect (*p<* 0.001, *R* ^2^ = 0.409; **Figure 4B**). We also found that although the relative abundance of saprotrophs influenced the mass loss of litter, their dissimilarity did not significantly affect the mean HFA effect (*p* = 0.292, *R* ^2^ = 0.369) (**Supplementary Figure S2**).

### Fungal community variation after glucose addition during later decomposition stages

**Figure 5:**
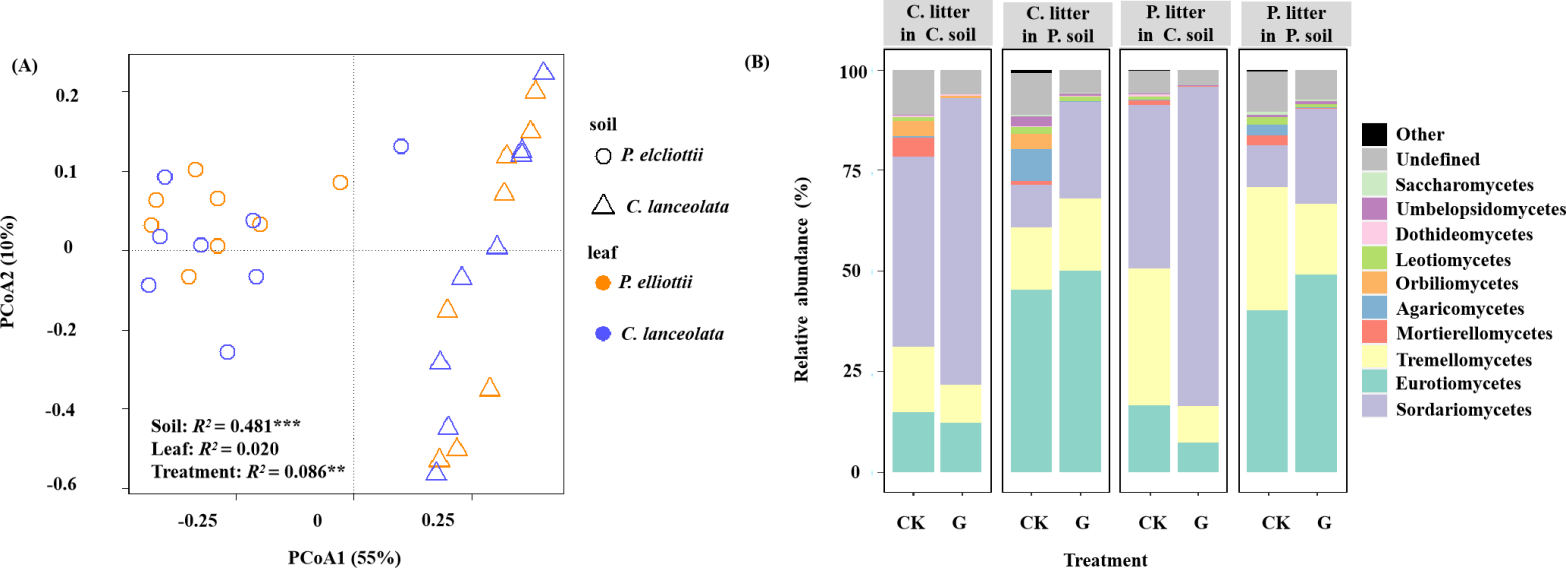
**(A)** Principal coordinate analysis (PCoA) plot of fungal community composition in soil under different treatments at the end of the 6th month. Soil sources are indicated by different symbols, while leaf litter species are indicated by different colors. **(B)** Relative abundance of the most abundant fungal classes in different soils, as in **(A)**. Different panels represent different soil sources and litter species, as indicated by the names in the panel. Within each panel, individual bars represent different treatments without (CK) or with (G) glucose addition at the end of the 4th month. The asterisks ** and *** indicate P < 0.01 and P < 0.001, respectively.

Analyses of the samples harvested after 6 months with and without glucose addition after 4 months, showed that the fungal sobs were significantly affected by treatment (*p<* 0.001), where the Shannon diversity was significantly influenced by both soil source (*p<* 0.001) and treatment (*p<* 0.001) (**Supplementary Table S6**). The soil source (*p<* 0.001, *R^2^* = 0.481) and treatment (*p* = 0.003, *R^2^* = 0.086) affected the fungal community structure, while litter species had no significant effect (**Figure 5A**; **Supplementary Table S7**). Glucose addition increased the relative abundance of the phylum Basidiomycota (*p<* 0.05; **Supplementary Table S8**).

### HFA after glucose addition in the late decomposition stages

**Figure 6:**
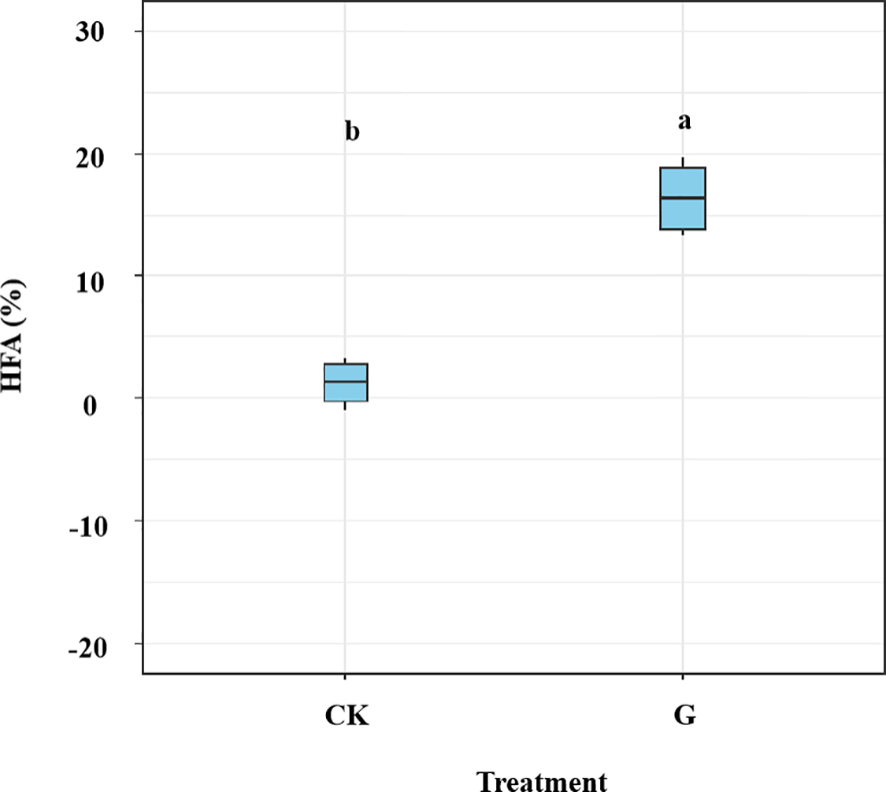
Effect of home-field advantage (HFA) on leaf litter decomposition of *Pinus elliottii* and *Cunninghamia lanceolata* in the different treatments at the end of the 6th month. The samples were prepared with (G) and without (CK) glucose addition after 4 months of decomposition. Data are shown as mean ± SE. Bars topped with different letters are significantly different at *p*< 0.05 using *t*-test.

**Figure 7:**
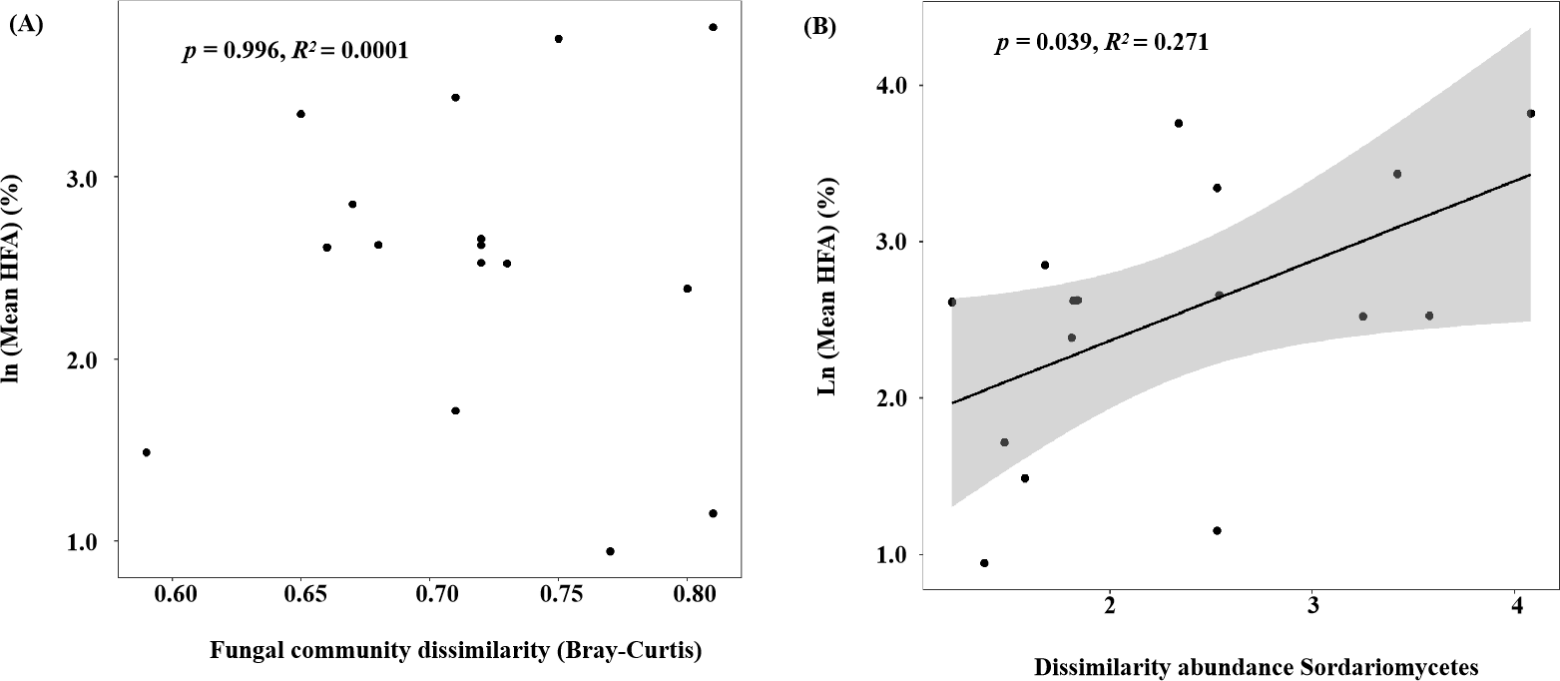
**(A)** Fungal community dissimilarity (Bray–Curtis) and **(B)** dissimilarity in the abundance of Sordariomycetes (calculated as the logarithm of the ratio of the relative abundance of Sordariomycetes in home and away soils) plotted against the mean home-field advantage (Mean HFA) effect. The samples were harvested at the end of the 6th month of decomposition with or without glucose addition at the end of the 4th month.

We found significant differences in HFA between samples with and without glucose addition (*p<* 0.05; **Figure 6**). The glucose addition led to a significant increase of 15.19% in the HFA effect (*p*< 0.05) during later decomposition stage. The mean HFA effect showed no significant correlation with fungal community dissimilarity between home and away sites, it was correlated with specific fungal taxa (i.e., the dissimilarity of class Sordariomycetes) (**Figure 7**). The dissimilarity in saprotrophs did not significantly affect the mean HFA effect (*p* = 0.74, *R* ^2^ = 0.008), however, the relative abundance of saprotrophs significantly influences the mass loss of litter (*p*< 0.001, *R* ^2^ = 0.45) (**Supplementary Figure S4**).

## Discussion

### Fungal community and HFA effects change over time

Consistent with hypothesis 1, the soil fungal community composition was influenced by soil source, litter species, time, and their interactions across the four harvest times. Time was the second-most important factor to explain the fungal community composition (**Figure 1**; **Supplementary Table S3**). Evidence suggests that the fungal community composition of decomposing litter underwent successional changes (Purahong et al., 2016). The litter loses cellulose and other simple C materials during the early decomposition stages, when the fast-growing *r*-strategists play a key role; however, the recalcitrant C compounds remain until the later litter decay stages (Berg, 2000; Hobbie et al., 2012; Berg, 2018; Kou et al., 2020). The phylum Ascomycota was dominant during the early decomposition stages but was gradually replaced by Basidiomycota in the later decomposition stages (**Supplementary Table S4**), consistent with previous findings (Voříšková and Baldrian, 2013). This is because of the different decomposition strategies, Ascomycota is copiotrophic (*r*-strategist) with high cellulolytic potential, while Basidiomycota (*K*-strategist) can secrete enzymes that degrade lignin and other recalcitrant polymers (Wang et al., 2020).

The fungal taxa mediate the chemical transformation of litter directly through their unique enzymatic ability and indirectly by competing with other decomposers for nutrients or energy (Wickings et al., 2012). The HFA effect consistently decreased over time when both *P. elliottii* and *C. lanceolata* leaf litter were considered (**Figure 3**). The variation in the HFA effect may be attributed to the decomposer community dissimilarity between home and away sites at different decomposition stages (Li et al., 2020). Greater differences in decomposer communities in home and away soils produce stronger HFA effects than in home and away soils with similar decomposer communities (Veen et al., 2019; Li et al., 2020). Our study found that the HFA effect was correlated with dissimilarity in the overall fungal composition (**Figure 4A**), inconsistent with the observations of Veen et al. (2019), as our destructive sampling across the four harvest periods, which may easily capture the variations in fungal community composition over time (**Figure 1**; **Supplementary Table S3**). Moreover, the HFA effect was strongly associated with the dissimilarity in Eurotiomycetes abundance between home and away soils (**Figure 4B**). Specifically, the enhanced decomposition of local litter on home soil by Eurotiomycetes increases the affinity between plant litter and fungal communities, thereby influencing the HFA effect. Eurotiomycetes are *r*-strategists, which tend to decrease in abundance during decomposition over time and produce enzymes that break down cellulose (Liu and Hall, 2004; Osono, 2007; Eichlerová et al., 2015). Saprotrophs showed a significant effect on litter decomposition, but their dissimilarity between home and away soils did not significantly influence the mean HFA effect (**Supplementary Figure S2**). It is well known that saprotrophic fungi play a pivotal role in litter decomposition, as they secrete various enzymes that break down complex organic compounds (Talbot et al., 2015; Fang et al., 2020). There were various taxa that possess saprotrophic properties; however, fungi are generally more efficient at decomposing litter that they frequently encounter in their home environment (Ayres et al., 2009a). Therefore, the influence of mean HFA is not due to the overall saprotrophic characteristic of fungi; instead, it is specific taxa that significantly related with the mean HFA.

Glucose addition affects the fungal community and HFA in later decomposition stages

Plant roots grow in the forest litter layer and continuously release fresh C into the soil as root exudates and root deposits (Wang et al., 2016), providing nutrients and energy for heterotrophic microorganisms (Kuzyakov et al., 2007; Kuzyakov, 2010). The glucose input, which provided a C source, modified the fungal community composition during the later decomposition stages (**Figure 5A**; **Supplementary Table S7**). Our result confirmed hypothesis 3, that organic inputs shape the development of fungal community composition (McTee et al., 2017). Irrespective of soil source and leaf litter species, compared with the addition of no glucose (CK) to soils, the glucose addition increased the relative abundance of the phylum Basidiomycota (*K*-strategist) (**Supplementary Table S8**). Fresh organic matter attracts fast-growing *r*-strategists in a short time. However, when the substrate is depleted, the *r*-strategists die and are dormant and are replaced by slow-growing *K*-strategists, which can secrete the enzymes to degrade complex polymers (e.g., Basidiomycota) (Fontaine et al., 2003; Kuzyakov, 2010).

Consistent with hypothesis 3, the inputs of glucose to the soil surface in contact with litter bags in the later decomposition stages strengthened the HFA effect (**Figure 6**). The result is similar to previous studies in literature, which showed that the local roots promoted the HFA effect, especially accelerating native litter decomposition by acting as real primers (Blagodatskaya and Kuzyakov, 2008; Tian et al., 2018). The input of fresh organic matter improves the activity and growth of previously nutrient-deprived microbes, which can exploit the new substrate (Fontaine et al., 2003; Clocchiatti et al., 2020). Root exudates of native plants can positively influence the function of local microorganisms that decompose native litter, thereby increasing the affinity of field and litter; however, these microorganisms do not have such an effect on non-native litter (Badri and Vivanco, 2009; Tian et al., 2018). The composition of glucose in home and away soils was the same in our study, however, glucose addition amplified the HFA effect (**Figure 6**). Glucose addition can strengthen the affinity between native litter and the home site. Although inputs of fresh organic matter activated dormant microorganisms in both the home and away sites, native litter in the home soil encountered more microorganisms familiar with self-decomposition than in the away soil, resulting in more rapid decomposition of litter in the home field. The mean HFA effect was correlated with the dissimilarity abundance of Sordariomycetes between the native and foreign soils in our study (**Figure 7**). Sordariomycetes, which perform various functions in the soil, such as breaking down recalcitrant organics, were the most abundant fungi in our study (**Figure 5B**) (Nguyen et al., 2016). Notably, glucose addition increased the relative abundance of the class Tremellomycetes (*p<* 0.05; **Supplementary Table S9**). However, the dissimilarity in this class did not affect the mean HFA of leaf litter of the two tree species. This is partially supported by empirical studies, which have found that repeated litter inputs influenced the fungal community composition but did not affect litter decomposition processes (Veen et al., 2021). This could be because a low monitored frequency does not fully capture the process of related fungal taxa affecting the HFA. Therefore, future studies should increase the frequency of sampling after glucose addition. In the sub-experiment of glucose addition, the dissimilarity in saprotrophs abundance between home and away soils exerted no significant influence on the mean HFA (**Supplementary Figure S4**), further demonstrating that the mean HFA effect is associated with specific fungal taxa.

Microorganisms, including bacteria and fungi, are the primary drivers of litter decomposition. They facilitate C and nutrient cycling by metabolizing complex organic compounds into simple forms through various enzymes (Palozzi and Lindo, 2018; Ayres et al., 2019a). Previous studies have revealed that fungal and bacterial keystone taxa, as well as saprotrophs, are involved in the decomposition dynamics (Fang et al., 2020; Zheng et al., 2021). Our results further showed that specific taxa influence the HFA effect. A previous study also demonstrated that specific decomposers not only accelerate litter decomposition but also enhances priming effect on soil organic matter (Di Lonardo et al., 2018). Interestingly, our study, on the other hand, found labile C input intensified the HFA effect at the later decomposition stage. Therefore, the HFA effects on litter mass loss and C pool dynamics may be larger than expected. In conclusion, our two-phase design study elucidates the dynamics of fungal communities and HFA effects over time, as well as their response to labile C inputs during litter decomposition, and illustrate the role of fungal community composition in this process. However, it is not without limitations. Specifically, our experiments were conducted under controlled laboratory conditions and focused on the fungal community, which excludes the potential influences from larger microbiota and fauna. Consequently, future research should integrate more microorganisms (such as bacteria) and other fauna (such as arthropods and earthworms) and be conducted in the field, in order to achieve a more comprehensive understanding of the role of soil organisms in litter decomposition.

## Conclusions

The fungal community composition and HFA effect on litter decomposition varied over time. In this process, the dissimilarity of the entire fungal composition, especially that of Eurotiomycetes, influenced the HFA effect. The addition of labile C to the soil surface directly contacting with litter bags during the later decomposition stages enhanced the HFA effect, which was mainly associated with specific fungal taxa (i.e., Sordariomycetes). Saprotrophs significantly affect the litter mass loss, however, their dissimilarity between home and away soils did not significantly affect the mean HFA. Future research should incorporate the effects of specific fungal taxa on the HFA effect, rather than indiscriminately integrating the entire fungal community. This study also highlighted the key role of underground root processes in litter decomposition, which affected the C and nutrient cycling. Therefore, in future research, consideration should be given to the priming effect of labile C on decomposition and the HFA effect at later decomposition stage.

## Data Availability

The sequencing data have been deposited in the NCBI database under the BioProject accession number PRJNA1232127.
