# Supplementary material for: Home-field advantage effect weakened over time but was strengthened by labile carbon input in later litter decomposition stage
Source: Front Plant Sci. 2025 Mar 14;16:1545311. doi: 10.3389/fpls.2025.1545311 (PMC11951264; doi:10.3389/fpls.2025.1545311)
Supplement: Supplementary file 1 [file DataSheet1.doc]

**Table S1** Initial chemistry properties of soil and leaf litter used in a microcosmic decomposition experiment. Data are presented as expressed as means ± standard error (*n* = 4). Different letters within the same row indicate significant differences in the chemical properties of soil or leaf litter between different tree species.

|  | ***P. elliottii*** | ***C. lanceolata*** |
| --- | --- | --- |
| Soil C (mg/g) | 35.33 ± 0.86^a^ | 19.65 ± 0.31^b^ |
| Soil N (mg/g) | 1.63 ± 0.05^a^ | 1.10 ± 0.01^b^ |
| Soil C: N | 21.84 ± 0.71^a^ | 17.36 ± 0.32^b^ |
| Leaf C (mg/g) | 501.72 ± 0.52^a^ | 487.59 ± 0.36^b^ |
| Leaf N (mg/g) | 5.72 ± 0.04^b^ | 9.15 ± 0.03^a^ |
| Leaf C: N | 87.79 ± 0.25^a^ | 53.31 ± 0.07^b^ |

**Table S2** The influence of soil source, litter species, harvest times and their interactions on fungal alpha diversity in the soils without glucose addition, as tested using ANOVA. Soil samples, without glucose addition, were analyzed across four harvest times.

|  | ***F*** | ***p*** |
| --- | --- | --- |
| **Sobs** |  |  |
| soil source | 45.992 | <0.001 |
| litter species | 2.045 | 0.159 |
| time | 4.015 | 0.013 |
| soil source × litter species | 12.001 | 0.001 |
| soil source × time | 7.201 | <0.001 |
| litter species × time | 2.535 | 0.068 |
| soil source × litter species × time | 0.713 | 0.549 |
|  |  |  |
| **Shannon diversity** |  |  |
| soil source | 2.031 | 0.161 |
| litter species | 5.570 | 0.022 |
| time | 4.465 | 0.008 |
| soil source × litter species | 0.607 | 0.440 |
| soil source × time | 1.305 | 0.284 |
| litter species × time | 1.848 | 0.151 |
| soil source × litter species × time | 4.642 | 0.006 |

**Table S3** The influence of soil source, litter species, harvest times, and their interactions on fungal community composition in the soils with no glucose addition, as tested using a PERMANOVA. The soil samples, without glucose addition, were analyzed across four harvest times.

|  | ***F*** | ***p*** | **R^2^** |
| --- | --- | --- | --- |
| soil source | 54.568 | < 0.001 | 0.351 |
| litter species | 12.439 | < 0.001 | 0.080 |
| time | 5.026 | < 0.001 | 0.097 |
| soil source: litter species | 3.997 | 0.005 | 0.026 |
| soil source: time | 3.06 | 0.002 | 0.059 |
| litter species: time | 2.061 | 0.017 | 0.040 |
| soil source: litter species: time | 2.009 | 0.020 | 0.039 |

**Table S4** Relative abundance (%) ± SE for dominant fungal phyla in all soils with no glucose addition over time. Different letters indicate significant differences in relative abundance among harvest period within a column (*p* < 0.05, Bonferroni post-hoc test).

| **Time** | **Ascomycota** | **Basidiomycota** | **Rozellomycota** | **Mortierellomycota** | **Mucoromycota** |
| --- | --- | --- | --- | --- | --- |
| **1 month** | 83.98±3.76^a^ | 12.89±3.54^b^ | 0.96±0.22^b^ | 0.98±0.34^a^ | 0.92±0.15^a^ |
| **2 months** | 76.31±4.02^ab^ | 16.11±3.48^ab^ | 2.42±0.34^a^ | 2.28±0.70^a^ | 2.30±0.43^a^ |
| **4 months** | 81.63±3.36^a^ | 13.73±3.04^b^ | 1.60±0.30^ab^ | 1.62±0.53^a^ | 1.18±0.20^a^ |
| **6 months** | 61.89±4.34^b^ | 30.16±4.61^a^ | 2.61±0.46^b^ | 2.32±0.64^a^ | 2.03±0.25^a^ |

**Table S5** Relative abundance (%) ± SE of eight fungal taxa at the class level with the highest relative abundance across samples with no glucose addition over time. Different letters indicate significant differences in relative abundance between harvest times (*p* < 0.05, Bonferroni post-hoc test). *P. elliottii*, *Pinus elliottii*; *C. lanceolata*, *Cunninghamia lanceolata*.

|  |  | **Sordariomycetes** | **Eurotiomycetes** | **Tremellomycetes** | **unclassified_p__Ascomycota** | **unclassified_p__Rozellomycota** | **Mortierellomycotina** | **unclassified_k__Fungi** | **Agaricomycetes** |
| --- | --- | --- | --- | --- | --- | --- | --- | --- | --- |
| ***P. elliottii* litter** | |  |  |  |  |  |  |  |  |
| *P. elliottii* soil | 1 month | 39.36±9.46^cdef^ | 53.20±7.20^ab^ | 2.67±0.96^d^ | 0.74±0.31^a^ | 1.37±0.72^abc^ | 0.51±0.29^a^ | 0.57±0.31^b^ | 0.45±0.23^a^ |
|  | 2 months | 41.79±3.62^cde^ | 41.40±6.38^bc^ | 2.57±0.27^d^ | 4.22±1.66^a^ | 2.04±0.16^abc^ | 2.90±1.17^a^ | 1.03±0.11^ab^ | 0.67±0.13^a^ |
|  | 4 months | 44.58±4.51^cde^ | 38.77±7.19^bcd^ | 3.44±0.28^d^ | 3.19±0.86^a^ | 2.97±0.22^abc^ | 3.22±1.16^a^ | 1.03±0.16^ab^ | 0.36±0.08^a^ |
|  | 6 months | 40.11±3.39^cde^ | 30.70±4.19^bcde^ | 10.47±3.25^cd^ | 4.25±0.59^a^ | 3.85±1.00^ab^ | 2.38±1.00^a^ | 1.92±0.41^ab^ | 2.63±1.72^a^ |
| *C. lanceolata soil* | 1 month | 9.41±2.53^g^ | 68.66±7.46^a^ | 14.15±3.55^cd^ | 1.49±0.38^a^ | 0.91±0.24^bc^ | 0.33±0.09^a^ | 1.23±0.32^ab^ | 1.29±0.61^a^ |
|  | 2 months | 16.42±1.74^defg^ | 34.75±2.59^bcde^ | 32.26±1.66^abc^ | 3.71±0.30^a^ | 2.70±0.23^abc^ | 1.89±0.57^a^ | 3.62±0.72^a^ | 0.77±0.12^a^ |
|  | 4 months | 10.42±1.18^fg^ | 52.37±6.17^ab^ | 27.26±3.56^abc^ | 3.64±0.58^a^ | 1.68±0.45^abc^ | 0.50±0.18^a^ | 1.86±0.33^ab^ | 0.28±0.06^a^ |
|  | 6 months | 16.43±4.37^defg^ | 34.05±5.27^bcde^ | 40.67±2.47^ab^ | 2.97±0.66^a^ | 1.24±0.53^abc^ | 1.28±0.39^a^ | 1.37±0.21^ab^ | 0.09±0.03^a^ |
| ***C. lanceolata* litter** | |  |  |  |  |  |  |  |  |
| *C. lanceolata* soil | 1 month | 18.64±1.22^defg^ | 42.62±6.24^abc^ | 27.79±4.94^abc^ | 2.54±0.95^a^ | 0.27±0.08^c^ | 2.73±0.91^a^ | 1.26±0.29^ab^ | 0.33±0.08^a^ |
|  | 2 months | 25.81±4.53^cdefg^ | 29.47±3.00^bcde^ | 24.42±6.28^abcd^ | 3.48±1.20^a^ | 1.21±0.42^abc^ | 1.35±0.51^a^ | 2.99±0.49^ab^ | 1.50±0.64^a^ |
|  | 4 months | 44.59±17.26^bcde^ | 21.21±3.72^cde^ | 18.25±9.08^bcd^ | 2.38±1.60^a^ | 0.26±0.15^c^ | 2.92±1.97^a^ | 1.27±0.81^ab^ | 0.11±0.07^a^ |
|  | 6 months | 14.86±2.48^efg^ | 16.18±4.49^cde^ | 47.46±10.33^a^ | 6.16±2.34^a^ | 2.00±1.11^abc^ | 4.67±2.02^a^ | 2.80±0.77^ab^ | 0.29±0.08^a^ |
| *P. elliottii* soil | 1 month | 82.56±6.21^a^ | 10.38±4.02^e^ | 1.18±0.32^d^ | 0.88±0.35^a^ | 1.22±0.32^abc^ | 0.33±0.15^a^ | 0.64±0.19^b^ | 0.79±0.56^a^ |
|  | 2 months | 54.70±5.22^abc^ | 17.91±1.80^cde^ | 4.42±0.74^d^ | 2.67±0.51^a^ | 3.92±0.69^a^ | 0.94±0.19^a^ | 2.57±1.14^ab^ | 3.17±1.53^a^ |
|  | 4 months | 73.93±5.24^ab^ | 12.70±1.25^de^ | 2.99±1.17^d^ | 1.08±0.36^a^ | 1.51±0.53^abc^ | 0.45±0.20^a^ | 0.79±0.28^b^ | 4.80±2.74^a^ |
|  | 6 months | 45.41±5.50^bcd^ | 16.96±2.79^cde^ | 10.55±4.51^cd^ | 5.04±1.55^a^ | 3.31±0.60^ab^ | 0.97±0.18^a^ | 2.05±0.30^ab^ | 6.30±3.06^a^ |

**Table S6 The influence of soil source, litter species, treatment (with and without glucose), and their interactions on fungal alpha diversity in the soils was tested using ANOVA. Glucose or nothing was added to the soil samples after decomposition for four months, and the samples were harvested after an additional two month of decomposition.**

|  | ***F*** | ***p*** |
| --- | --- | --- |
| **Sobs** |  |  |
| soil source | 4.156 | 0.053 |
| litter species | 2.681 | 0.115 |
| treatment | 99.033 | <0.001 |
| soil source × litter species | 1.909 | 0.180 |
| soil source ×treatment | 4.083 | 0.055 |
| litter species ×treatment | 0.346 | 0.562 |
| soil source × litter species ×treatment | 4.385 | 0.048 |
|  |  |  |
| **Shannon diversity** |  |  |
| soil source | 20.898 | <0.001 |
| litter species | 0.354 | 0.557 |
| treatment | 46.918 | <0.001 |
| soil source × litter species | 7.987 | 0.010 |
| soil source ×treatment | 14.121 | 0.001 |
| litter species ×treatment | 0.360 | 0.555 |
| soil source × litter species ×treatment | 5.172 | 0.033 |

**Table S7** The influence of soil source, litter species, treatment (with and without glucose), and their interactions on fungal community composition in the soils, as tested using PERMANOVA. Glucose or nothing was added to the soil samples after decomposition for four months, and sample were harvested after another two months.

| **Factor** | **F** | ***p*** | **R^2^** |
| --- | --- | --- | --- |
| **soil source** | 34.644 | 0.481 | 0.001 |
| **litter species** | 1.466 | 0.021 | 0.204 |
| **treatment** | 6.202 | 0.086 | 0.003 |
| **soil source: litter species** | 0.736 | 0.010 | 0.492 |
| **soil source: treatment** | 2.936 | 0.041 | 0.041 |
| **litter species: treatment** | 1.281 | 0.018 | 0.253 |
| **soil source: litter species: treatment** | 0.719 | 0.010 | 0.536 |

**Table S8** Relative abundance (%) ± SE of dominant fungal phyla in soils after decomposition for six months. The soil samples were treated with (G) or without (CK) glucose addition after decomposition for four months and harvested after another two months. Different letters indicate significant differences in relative abundance among harvest periods within a column (*p* < 0.05, Bonferroni post-hoc test).

| **Treatment** | **Ascomycota** | **Basidiomycota** | **Rozellomycota** | **Mortierellomycota** | **Mucoromycota** |
| --- | --- | --- | --- | --- | --- |
| **CK** | 61.89±4.34^a^ | 30.16±4.61^b^ | 2.61±0.46^a^ | 2.32±0.64^a^ | 0.88±0.45^a^ |
| **G** | 49.14±5.72^b^ | 49.57±7.61^a^ | 0.46±0.09^b^ | 0.18±0.05^b^ | 0.36±0.10^a^ |

**Table S9** Relative abundance (%) ± SE of eight dominant fungal taxa at the class level across samples with (G) or without (CK) glucose addition after decomposition for four months and harvested after another two months. Different letters indicate significant differences in the relative abundance between treatment combinations within a column (*p* < 0.05, Bonferroni post-hoc test).

| **Treatment** | **Sordariomycetes** | **Eurotiomycetes** | **Tremellomycetes** | **unclassified_p__Ascomycota** | **unclassified_p__Rozellomycota** | **Mortierellomycotina** | **unclassified_k__Fungi** | **Agaricomycetes** |
| --- | --- | --- | --- | --- | --- | --- | --- | --- |
| **CK** | 29.20±3.98^a^ | 24.06±2.95^a^ | 27.29±5.13^b^ | 4.60±0.72^a^ | 2.60±0.46^a^ | 2.32±0.64^a^ | 2.03±0.25^a^ | 2.15±0.90^a^ |
| **G** | 29.68±5.84^a^ | 13.75±1.65^b^ | 49.49±7.62^a^ | 4.90±0.60^a^ | 0.46±0.09^b^ | 0.18±0.05^b^ | 0.27±0.04^b^ | 0.07±0.02^b^ |

**Figure S1** (A) Mass remaining (%) of *Pinus elliottii* (*P. elliottii*) and *Cunninghamia lanceolata* (*C. lanceolata*) litter with no glucose addition over time. (B) Mean home-field advantage (Mean HFA) for the decomposition of *Pinus elliottii* (*P. elliottii*) and *Cunninghamia lanceolata* (*C. lanceolata*) between home and away soils with no glucose addition over time.

**Figure S2** (A) The relationship between the relative abundance of Saprotroph and the mass loss of decomposing litter is shown. (B) The dissimilarity in the abundance of Saprotroph (calculated as the logarithm of the ratio of the relative abundance of Saprotroph in home and away soils) plotted against the log-transformed mean home-field advantage (Mean HFA) effect. Sample data from the soils without glucose addition were collected at four harvest times (after 1, 2, 4, and 6 months of decomposition).

**Figure S3** The mean home-field advantage (Mean HFA) for the decomposition of *Pinus elliottii* (*P. elliottii*) and *Cunninghamia lanceolata* (*C. lanceolata*) between home and away soils at the end of six months. Glucose or nothing was added to the soil samples after decomposition for four months, and samples were harvested after another two months.

**Figure S4** (A) The relationship between the relative abundance of Saprotroph and the mass loss of decomposing litter is shown. (B) The dissimilarity in the abundance of Saprotroph (calculated as the logarithm of the ratio of the relative abundance of Saprotroph in home and away soils) plotted against the log-transformed mean home-field advantage (Mean HFA) effect. The samples were collected at the end of the 6th month of decomposition, following the addition of glucose or no addition at the end of the 4th month.
